# Time Processing and Motor Control in Movement Disorders

**DOI:** 10.3389/fnhum.2016.00631

**Published:** 2016-12-12

**Authors:** Laura Avanzino, Elisa Pelosin, Carmelo M. Vicario, Giovanna Lagravinese, Giovanni Abbruzzese, Davide Martino

**Affiliations:** ^1^Department of Experimental Medicine, Section of Human Physiology and Centro Polifunzionale di Scienze Motorie, University of GenoaGenoa, Italy; ^2^Department of Neuroscience, Rehabilitation, Ophthalmology, Genetics and Maternal Child Health, University of GenoaGenoa, Italy; ^3^School of Psychology, University of TasmaniaHobart, TAS, Australia; ^4^Wolfson Centre for Clinical and Cognitive Neuroscience, School of Psychology, Bangor UniversityBangor, UK; ^5^International Parkinson's Centre of Excellence, King's College and King's College Hospital, Denmark Hill CampusLondon, UK; ^6^Queen Elizabeth Hospital, Woolwich, Lewisham, and Greenwich NHS TrustLondon, UK

**Keywords:** timing and time perception, movement disorders, neural pathways, motor control, rehabilitation

## Abstract

The subjective representation of “time” is critical for cognitive tasks but also for several motor activities. The neural network supporting motor timing comprises: lateral cerebellum, basal ganglia, sensorimotor and prefrontal cortical areas. Basal ganglia and associated cortical areas act as a hypothetical “internal clock” that beats the rhythm when the movement is internally generated. When timing information is processed to make predictions on the outcome of a subjective or externally perceived motor act, cerebellar processing and outflow pathways appear to be primarily involved. Clinical and experimental evidence on time processing and motor control points to a dysfunction of the neural networks involving basal ganglia and cerebellum in movement disorders. In some cases, temporal processing deficits could directly contribute to core motor features of the movement disorder, as in the case of bradykinesia in Parkinson's disease. For other movement disorders, the relationship between abnormal time processing and motor performance is less obvious and requires further investigation, as in the reduced accuracy in predicting the temporal outcome of a motor act in dystonia. We aim to review the literature on time processing and motor control in Parkinson's disease, dystonia, Huntington's disease, and Tourette syndrome, integrating the available findings with current pathophysiological models; we will highlight the areas in which future explorations are warranted, as well as the aspects of time processing in motor control that present translational aspects in future rehabilitation strategies. The subjective representation of “time” is critical for cognitive tasks but also for motor activities. Recently, greater attention has been devoted to improve our understanding of how temporal information becomes integrated within the mechanisms of motor control. Experimental evidence recognizes time processing in motor control as a complex neural function supported by diffuse cerebral networks including cortical areas, cerebellum, and other subcortical structures (Ivry and Spencer, 2004; Coull and Nobre, 2008). Timing is an essential component of motor control primarily within two types of motor tasks: (i) when producing sequential rhythmic movements or sustained movements of a definite duration (*explicit* timing); (ii) when the temporal information is used implicitly, such as when coordinating our movements to those of moving objects or individuals within the external environment (*implicit* timing). In this review, we will provide a brief description of the neural network supporting motor timing focusing only on instrumental information to explain the link between timing and motor control in movement disorders. Then we will review available data on motor timing in Parkinson's disease, dystonia, Huntington's disease, and Tourette syndrome, and discuss how this body of evidence integrates with the available information on the pathophysiology of these movement disorders. Finally, we will discuss the translational aspects of the explored neural mechanisms with respect to future rehabilitation strategies.

## The neural network of motor timing

### Explicit timing

When performing explicit timing tasks, we make an explicit use of temporal information (e.g., estimates of the duration of different stimuli or of their inter-stimulus intervals [ISI]) in order to represent precise temporal durations through a sustained or periodic motor act (Coull and Nobre, 2008). Functional magnetic resonance imaging (fMRI) (Bengtsson et al., 2004, 2005; Lewis et al., 2004; Jahanshahi et al., 2006; Bueti et al., 2008) and non-invasive brain stimulation (Koch et al., 2003; Dusek et al., 2011; Vicario et al., 2013) studies of motor timing have consistently identified brain regions crucial to time processing, in particular supplementary motor area (SMA), basal ganglia (BG), cerebellum and right-inferior frontal and parietal cortices.

**Figure 1:**
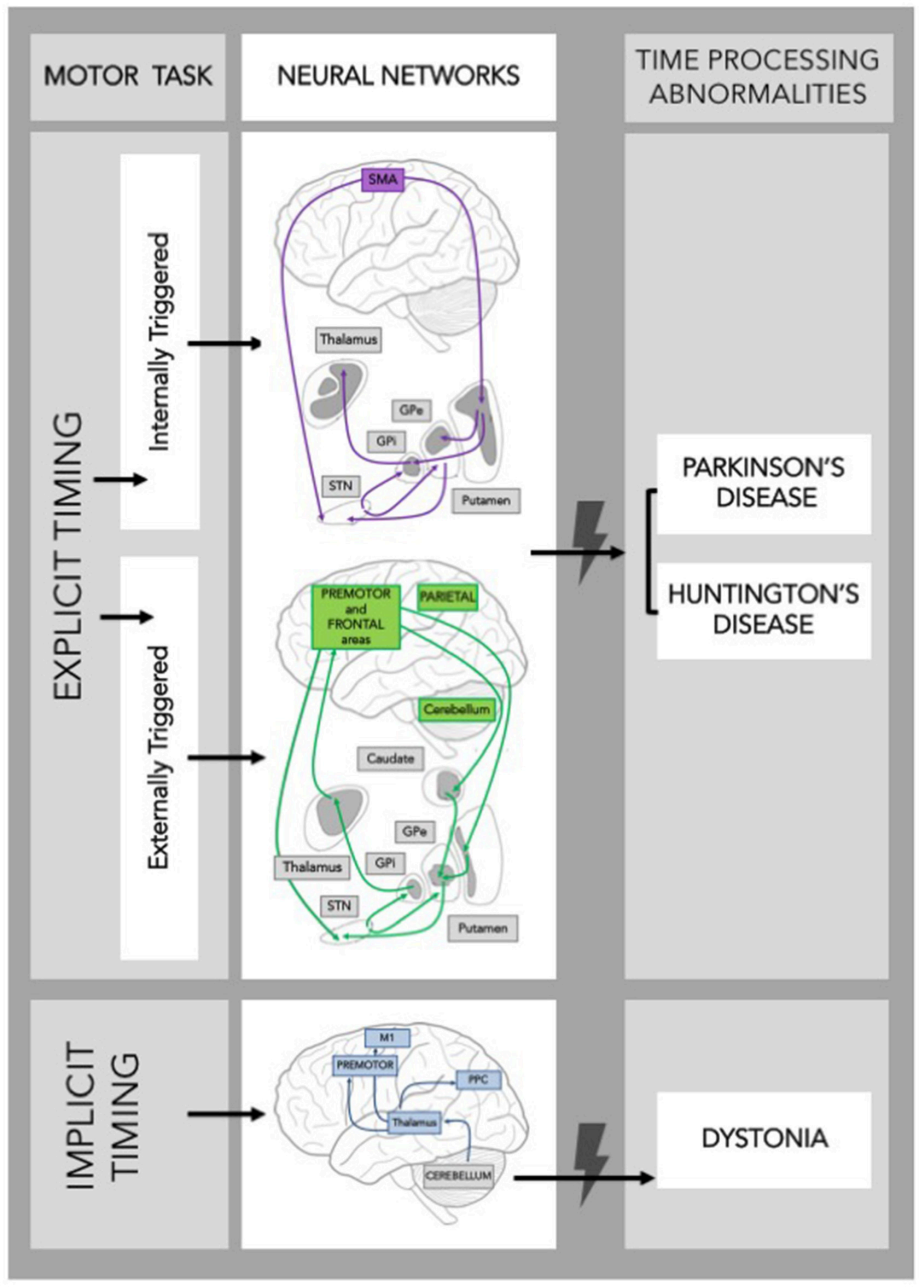
**Schematic frame of the neural networks supporting timing during a motor task**. Neural networks involved in explicit and implicit timing and their possible contribution to time processing abnormalities in Movement Disorders are shown. GPe, Globus Pallidus external; GPi, Globus Pallidus internal; STN, SubThalamic Nucleus; SMA, Supplementary Motor Area; M1, Primary Motor Cortex; PPC, Posterior Parietal Cortex.

The synchronization-continuation paradigm has been frequently used to study the neural substrates of explicit motor timing. This paradigm allows for the study of both externally triggered and internally triggered movements as it involves: (i) a synchronization phase, in which subjects are asked to tap in synchrony with a train of tones separated by a constant ISI, and (ii) a continuation phase, in which subjects are requested to continue tapping at the previous rate in the absence of the auditory cue. Using fMRI paradigms, Rao et al. (1997) showed that the continuation (internally triggered) phase, but not the synchronization (externally triggered) phase, activates the SMA, the left-caudal putamen, and the left ventrolateral thalamus. These findings support the existence of a hypothetical “internal clock” that beats the rhythm when the movement is internally generated (Pastor et al., 1992b; Meck and Benson, 2002; François-Brosseau et al., 2009), and involves the sensorimotor circuit of the BG (Figure 1). Indeed, it has been proposed that several parallel, segregated loops through the BG connect back to distinct cortical areas (namely motor, associative, and limbic network) (Alexander et al., 1986). The sensorimotor loop connects the motor areas in the cortex with the caudolateral striatum territories playing a role in stimulus-response habitual control (Redgrave et al., 2010).

The role of the BG in timing was further substantiated by demonstrating that they are activated during motor, but also perceptual, time processing tasks (Bueti et al., 2008) involving both sub-second and supra-second temporal intervals (Jahanshahi et al., 2006). Noteworthy, the associative basal ganglia circuit is particularly active during externally triggered tasks (Figure 1).

The cerebellum, instead, is more often activated (i) during motor than perceptual explicit timing tasks (Bengtsson et al., 2005; Jahanshahi et al., 2006; Bueti et al., 2008), (ii) when a synchronization to an external rhythm is required (Del Olmo et al., 2007), and (iii) when processing involves sub-second rather than supra-second intervals (Lewis and Miall, 2003a,b).

### Implicit timing

Timing is implicit when temporal information can optimize performance on a non-temporal task. The processing of time-dependent features of a movement is crucial in predicting whether the outcome of the movement will be consistent with its ultimate goal, or will result in an execution error. When temporal information is processed to predict the outcome of self-executed or externally-driven movements, cerebellar outflow pathways are primarily involved. Indeed, cerebellar networks optimize self-executed actions by recalibrating predictions, capturing the sensory consequences of the same actions (Bell et al., 2008; Synofzik et al., 2008; Izawa et al., 2012). When subjects use temporal information inherent to the spatial-temporal trajectory of a dynamic visual stimulus to predict its final position, fMRI studies revealed activation in the left inferior parietal cortex (Assmus et al., 2005), sensorimotor regions of the premotor and parietal cortices (Field and Wann, 2005), and cerebellum (O'Reilly et al., 2008). We recently showed that the cerebellum is engaged when temporal information is processed to predict the temporal outcome of a motor act (Avanzino et al., 2015a). We developed an *ad hoc* task in which participants were required to observe a movement in a video and then predict the end of the same movement (Avanzino et al., 2013, 2015a; Martino et al., 2015). Crucially, a few seconds after its onset, the video was darkened for a given time interval, thus the task could be performed only by extrapolating time-related features of observed motion sequence. By this task, we have shown that inhibiting the activity of the lateral cerebellum with 1 Hz-repetitive transcranial magnetic stimulation induced a deterioration of timing performance selectively when subjects were asked to estimate the duration of a body segment movement (handwriting) and not of a movement involving an inanimate object (Avanzino et al., 2015a). This finding may suggest that cerebellar sub-regions are specifically involved in processing temporal features of movements belonging to the human motor repertoire.

## Evidence on motor timing abnormalities in movement disorders (Box 1)

### Parkinson's disease

The notion that the mechanisms underlying time processing are abnormal in PD has been suggested by some pivotal observations. Pastor et al. (1992a,b) reported that the accuracy in timing of repetitive alternating wrist movements, time estimation and time reproduction on a verbal task were impaired in PD patients “off” medication, indicating timing mechanisms abnormalities in PD already at a perceptual level and possibly related to dysfunction of an “internal clock” that is modulated by dopamine.

Box 1 **Brief description of the selected scientific papers on time processing and motor control in Parkinson's disease, dystonia, Huntington's disease, and Tourette syndrome.**

**Table:** 

**Condition**	**Explicit motor timing (Paced and self-paced finger tapping [PFT and S-PFT]; repetitive wrist movements; time reproduction tasks)**	**Explicit perceptual timing (Time production/estimation and time discrimination tasks)**	**Implicit motor timing (Temporal expectation tasks; in *musician's dystonia*: time analysis of performed musical scales or finger tapping performance on keyboard)**	**Implicit perceptual timing (Temporal expectation tasks)**
Parkinson's disease	• Reduced accuracy for both sub-second and supra-second intervals • Inconsistent changes in performance variability (increased on PFT tasks, decreased with time reproduction) • Inconsistent effects of dopaminergic medication on performance	• Reduced accuracy for suprasecond intervals (evidence of improvement with dopaminergic medication, which needs to be replicated)	• No obvious changes	• Not explored
Huntington's disease	• Reduced accuracy and increased performance variability observed more consistently for supra-second intervals, with changes observed in symptomatic and pre-symptomatic subjects (in the latter, performance changes increase as estimated years to onset decrease)	• Reduced accuracy for suprasecond intervals	• Not explored	• Not explored
Dystonia	• No obvious changes on PFT tasks	• No obvious changes	• In musician's dystonia, reduced accuracy of the affected hand • Variable finger tapping performance on keyboard, depending on finger affected	• In writer's cramp and cervical dystonia, reduced accuracy on temporal prediction of hand motion, but not of inanimate object motion
Tourette syndrome	• Reduced accuracy on time reproduction tasks for supra-second intervals, with performance variability influenced by dopamine D2 receptor blockers	• No obvious changes	• Not explored	• Not explored

Afterwards, impaired time reproduction was confirmed during paced finger tapping in PD (O'Boyle et al., 1996; Elsinger et al., 2003) and assessed in “on” and “off” conditions (Jones et al., 2008). Using the synchronization-continuation task to investigate both *de novo* and treated PD subjects, Jones et al. (2011) showed that treated PD patients tapped ahead of the beat at the 250 ms rate, respect to *de novo* and healthy subjects without any difference between “off” and “on” conditions. This observation, resembling the clinical phenomenon of festination that typically occurs in PD for fast repetitive movements, suggested an influence of either chronic pharmacological treatment or disease progression. Altogether, the continuation phase highlighted major differences likely reflecting the difficulties of PD subjects with internally generated movements.

More recently, using a modified version of the synchronization-continuation paradigm with a motor imagery task, we showed that PD patients exhibited a selective deficit in motor timing for internally triggered sequential movements separated by a supra-second interval, and that this deficit was better explained by a defect of motor planning (Avanzino et al., 2013). Bienkiewicz and Craig (2015) showed that abnormalities in sensorimotor synchronization in PD are not limited to finger tapping task but rather extend in the context of a beat interception task based on aiming movements. The type of task required prospective motor control (i.e., coupling movement to neural based dynamic information that helps anticipate when the beat is going to sound) suggesting that BG circuitry might undermine also the temporal prediction ability.

Overall, the motor timing abnormalities observed in PD indicate that the BG are mainly involved in explicit timing mechanisms (see also Martinu et al., 2012). Accordingly, a recent study showed that the implicit timing of salient events seems largely unaffected by PD (de Hemptinne et al., 2013). It is worthwhile to underline that the tight interaction between difficulties with cognitive timing and spatio-temporal control of movement emerges not only from experimental findings, but also from the clinical aspects of PD. Bradykinesia (slowness of movement initiation and execution) is particularly evident for internally generated sequential movements (McIntosh et al., 1997; Heremans et al., 2012), commonly occurs in gait and can manifest as slow shuffling strides, an accelerating gait, or highly variable and random stride times (Baltadjieva et al., 2006; Almeida et al., 2007).

Related to the neural network supporting motor timing abnormalities in PD, evidence demonstrated decreased activation within the sensorimotor cortex, cerebellum, and medial premotor system in PD patients compared to controls during paced finger tapping (Elsinger et al., 2003). Dopamine supplementation did not improve task performance, but partially “normalized” brain activation patterns. In a PET study PD patients presented a significantly enhanced activation of the cerebellum, thalamus and substantia nigra reticulata during a synchronization-continuation task, compared to healthy subjects (Jahanshahi et al., 2010). Moreover, pallidal over-activation and cortical regions under-activation were observed in PD subjects in the “off” state, but switching to the “on” state was associated with increased striato-frontal functional connectivity. This study and others (Husárová et al., 2013, 2014) supported the idea that motor timing networks are modulated by dopaminergic stimulation and that PD patients activate an alternative timing network relying more on cerebellar activation than healthy controls. Summarizing the behavioral and imaging evidence on the influence of dopaminergic medication on motor timing, so far results are mixed and therefore conclusions are unclear. The role of the BG oscillatory activity in motor timing has been evidenced in a group of 12 PD patients implanted for subthalamic nucleus deep brain stimulation (STN-DBS) (Wojtecki et al., 2011). Supra-second time reproduction performance was significantly improved during “on” stimulation (130-Hz) compared to no stimulation, whereas milliseconds timing was not affected.

### Huntington's disease

Huntington's disease (HD) is a hereditary neurodegenerative disorder, caused by an expansion of CAG triplet repeats in the *Huntingtin* gene on chromosome 4. The monogenic etiology of HD allowed investigating timing in both symptomatic and pre-symptomatic individuals. Explicit timing abilities progressively deteriorate in mutation carriers as they approach clinical disease onset. Early-moderate HD patients exhibited marked irregularity of tapping rates on the synchronization-continuation paradigm (Freeman et al., 1996). In pre-symptomatic HD mutation carriers, reproduction of supra-second intervals accuracy decreases with estimated time to onset, Hinton et al. (2007) and Rowe et al. (2010). A self-paced variant of the same tapping task was also used to investigate the relationship between timing performance and disease progression. Performance on self-paced finger tapping was associated with disease progression (Michell et al., 2008; Tabrizi et al., 2009; Bechtel et al., 2010), showing high sensitivity in separating mutation carriers from non-carriers already from the earliest stages (Tabrizi et al., 2009; Bechtel et al., 2010). A similar pattern of timing deterioration was observed on non-rhythmic motor reproduction of supra-second intervals, which became less precise as disease onset approached and progressed further as disease advanced (Beste et al., 2007; Wild-Wall et al., 2008; Rao et al., 2014).

Functional magnetic resonance imaging (fMRI) confirmed that performance on paced finger tapping was associated with reduced neural activation in subcortical regions (putamen, caudate, thalamus) in pre-symptomatic HD mutation carriers closer to disease onset (Zimbelman et al., 2007). This subgroup of pre-symptomatic patients under-activated the SMA, left-anterior insula and right-inferior frontal gyrus during the finger tapping task. Conversely, those far from onset exhibited more efficient compensatory activation of other brain areas (sensorimotor cortex, medial frontal cortex and cerebellum) (Zimbelman et al., 2007).

Overall, the progressive involvement of cortico-basal ganglia sensorimotor and associative loops in HD patients is reflected by a similarly progressive timing deficit that is more selective for explicit, cognitive-controlled timing tasks rather than implicit timing tasks.

### Dystonia

Dystonia is a network movement disorder associated with maladaptive plasticity of motor output coupled to disordered sensorimotor integration. Dysfunction in both cortico-basal ganglia and cerebello-thalamo-cortical networks is believed to give rise to the involuntary tonic and phasic muscle contractions that characterize dystonia. Timing has been assessed mainly in task-specific forms of dystonia, triggered by complex movements like musical playing or writing. Kinematic analyses of scales or finger tapping performed on a digital piano by pianists with dystonia showed inaccuracies in tone and interval duration and rhythmic inconsistency (Jabush et al., 2004; Furuya and Altenmüller, 2013). This may represent a mere consequence of the motor overflow during musical performance, since part of these measures improved with botulinum toxin therapy (Jabush et al., 2004; Furuya et al., 2014). Further, explicit timing performance in these patients appeared similar to control subjects (Van Der Steen et al., 2014), suggesting that direct extrapolation of temporal properties while performing timed movements may be preserved in dystonia. On the other hand, we showed that patients with either task-specific (writer's cramp) (Avanzino et al., 2013) or non-task-specific (cervical) dystonia (Martino et al., 2015) are less accurate in predicting the temporal outcome of a visually perceived movement, and that this is observed for human body, but not inanimate object, motion. This supports the view that patients with dystonia manifest subtle differences in extrapolating temporal properties during the perception of hand/arm movements. Such deficit may be linked to an abnormal internal model of motor commands reflecting dysfunction of cerebellar outflow pathways. The selective implicit timing task abnormalities support the notion that dystonia is a broader network disorder, in which the crucial nodes are located not only in the BG and the sensorimotor cortex, but also in the cerebellum.

### Tourette's syndrome

Tourette's syndrome (TS) is associated with the abnormal maturation of cortico-basal ganglia loops. Timing abilities are underexplored in TS. In an early report, discrimination between different time intervals in untreated patients was comparable to healthy subjects (Goldstone and Lhamon, 1976). More recently Goudriaan et al. (2006) reported that time estimation and reproduction of time intervals spanning from 2 to 20 s did not differ between TS and healthy adults. However, these authors measured time intervals using a manual stopwatch, which is poorly accurate. A study by Vicario et al. (2010) has documented higher accuracy in the processing of supra-second intervals in TS children with lower tic severity, compared to age-matched healthy volunteers (Vicario et al., 2010). This result was explained by suggesting the existence of an effective compensatory modulation phenomenon, in correspondence of the prefrontal cortex, leading to better tic inhibition and increased precision in explicit processing of supra-second time intervals. Dopaminergic modulation seems also relevant to temporal discrimination in TS patients, as dopamine receptor blockers like pimozide may improve patients' variability on this task (Vicario et al., 2016).

Overall, TS has been investigated mainly for perceptual aspects of timing, revealing greater accuracy of temporal discrimination of supra-second intervals associated with lower tic severity. As already suggested for other behavioral abnormalities in TS (Mueller et al., 2006; Jackson et al., 2007), this behavioral gain appears to be more linked to adaptive mechanisms facilitating tic suppression rather than to core pathogenic mechanisms.

## Translational aspects in rehabilitation strategies

Knowledge on abnormalities in time processing for motor control has been already applied to plan rehabilitative strategies in movement disorders. For PD, rehabilitation approaches to compensate defective internal rhythm generation by the BG have been designed. Rhythm-related interventions (such as external sensory cueing or music) have been extensively used in motor rehabilitation. Most studies showed that gait impairment in PD could be significantly improved by rhythmic (auditory) stimulation (Lim et al., 2005, 2010) or musically cued gait training (Benoit et al., 2014). Rhythmic external cueing was also found to improve upper limb movements of PD patients (Vercruysse et al., 2012), and even assist motor learning (Nieuwboer et al., 2009). However, synchronization with a fixed-tempo may be critical in PD subjects (Bienkiewicz and Craig, 2015; Young et al., 2016) and the effect may be very limited in time since learning may be specific to cued rather than non-cued performance. Thus, novel strategies have been recently proposed involving interactive systems providing adaptive rhythmic stimulations (Hove and Keller, 2015) video (Pelosin et al., 2013) or mirror visual feedback training (Bonassi et al., 2016).

The motor timing abnormalities observed in hyperkinetic disorders might have a role in disease monitoring (HD), development of novel rehabilitative strategies (dystonia), or monitoring of existing interventions (tic disorders). Self-paced finger tapping and time reproduction of supra-second intervals have proved to be sensitive sub-clinical markers of disease progression in HD, and their application to experimental trials of disease-modifying treatments is potentially very rewarding.

Several experimental approaches aimed to promote the re-organization of sensory-motor regions to improve motor control and symptoms of dystonia (Avanzino et al., 2015b). Available information in dystonia shows reduced accuracy in extrapolating temporal properties during the perception of body movements. Sensory-motor integration is central to new physiological models of motor control, whereby motor outflow generated by the primary motor cortex is associated with the generation of efferent copies that allow anticipating the sensory consequences of the motor program (Perruchoud et al., 2014). This anticipatory ability could be crucial to motor performance, and could be influenced also by perceptual experiences of internal and external movements. Within this theoretical framework, temporal expectations tasks applied to external or internal movements might be exploited as rehabilitative strategies to improve the ability to anticipate “on-line” the sensory consequences of motor output in dystonia.

Finally, cognitive control over tics is an important component of behavioral treatment strategies (Piacentini et al., 2010; Van de Griendt et al., 2013). Predictors of response to behavioral treatments for tics are still uncertain, and performance on tasks assessing cognitive control of motor actions may represent a useful predictive marker. Interestingly, preliminary studies of time processing in TS suggest a possible link between cognitively controlled tasks and timing, indicating that time processing tasks might be considered in future studies on cognitive predictors of response to behavioral treatment (Jung et al., 2013; Vicario et al., 2013).

## Author contributions

LA, GA, and DM contributed to literature search, writing of preliminary draft, text revision, and finalization. EP, CV, and GL contributed to literature search, writing of preliminary draft.

### Conflict of interest statement

The authors declare that the research was conducted in the absence of any commercial or financial relationships that could be construed as a potential conflict of interest.
